# Transient erythroblastopenia of childhood after COVID-19 infection: a case report

**DOI:** 10.1186/s13052-024-01700-2

**Published:** 2024-07-29

**Authors:** Giulio Rivetti, Fabio Giovanni Abbate, Marialaura Longobardi, Maria Maddalena Marrapodi, Francesca Lanzaro, Martina Di Martino, Fara Vallefuoco, Velia D’Angelo, Maddalena Casale, Immacolata Tartaglione, Silverio Perrotta, Domenico Roberti

**Affiliations:** Department of Woman, Child and General and Specialized Surgery, University of Campania “Luigi Vanvitelli”, Via Luigi De Crecchio, Naples, 80138 Italy

**Keywords:** COVID-19, TEC, MIS-C

## Abstract

**Background:**

Transient erythroblastopenia of childhood (TEC) is an acquired, self-limited pure red cell aplasia that usually occurs in children 4 years old and younger. This clinical condition has been priorly described to be linked to numerous viral and immunologic mechanisms. COVID-19, caused by the coronavirus SARS-CoV-2, was initially discovered in China in December 2019. The disease quickly spread worldwide, resulting in pandemic.

**Case Presentation:**

This manuscript reports a new clinically relevant condition associated to COVID-19, describing a child with clinical and biochemical signs of Pure Red Blood cells aplasia and complete absence of erythroblasts at the bone marrow needle aspiration with signs of erythrophagocytosis, resembling morphological signs such as in hemophagocytic lymphohistiocytosis (HLH), temporally associated to SARS-CoV-2 infection.

**Conclusion:**

This report highlights a newly described continuum laboratory and clinical spectrum of immune/hematological dysregulations secondary to SARS-CoV-2. SARS-CoV‐2 infection-linked TEC has never been described in literature, but, according to our findings, should be considered in all the patients with transient erythroblastopenia without congenital red blood cell abnormalities and serology negative for major infections associated with TEC. This condition must be considered in the same spectrum of MIS-C and the inter-links among the two clinical manifestations, as well as a potential interdependence among them, should be considered in the future.

## Background

Transient erythroblastopenia of childhood (TEC) is an acquired, self-limited pure red cell aplasia that usually occurs in children 4 years old and younger [1]. It is characterized by a hemoglobin level at least 2 SDs below normal and a low reticulocyte count in absence of evidence of alternative causes of anemia in an otherwise normocellular bone marrow with lack of erythroid precursors [1]. The anamnestic data of a viral infection (such as Parvovirus B19, Epstein-Barr virus, cytomegalovirus, human herpes virus type 6, and echovirus) preceding the anemia and a significantly reduced quantity of erythroblasts in the bone marrow without underlying congenital red blood cell abnormalities are typical of TEC [1]. Most of the time, within two weeks from the diagnosis, hematopoiesis’ processes recover, and, within two months, a complete normalization of blood counts can be displayed. In fact, red blood cells transfusion is usually reserved for cases where there is hemodynamic instability, exercise intolerance, or altered mental status [2]. SARS-CoV‐2, since 2019, has caused more than 200 million respiratory infections, inducing a systemic innate and adaptive immune activation; alongside the respiratory symptoms, Covid-19 has been shown to induce also hematologic disorders such as thrombocytopenia and thrombosis [3], lymphopenia [4], neutropenia [5], disseminated intravascular coagulation and Covid-19 associated coagulopathy [6] but it was never associated to TEC. In healthy children, hematological findings during COVID-19 infection seem to slightly differentiate from adults [7]. The most common hematological abnormality described in the literature is indeed leukopenia [8], although the majority of the described pediatric patients with COVID-19 infection have shown normal or high white blood cells together with uncommon abnormalities of red blood cells and platelets. Anemia and thrombocytopenia/hypercoagulability has been mostly described in children affected by a severe multisystem inflammatory syndrome (MIS) associated with SARS-CoV-2 [9]. Sporadic cases of specific alterations in otherwise healthy children have, however, been described such as delayed immune thrombocytopenia [10] as well as bone marrow aplasia such as in a report published in 2022 where Šimić et al. documented a case of normocytic anemia in association with COVID-19 infection [11]. In this report, a two-and-a-half-year-old girl presented with severe normocytic anemia concurrent with COVID-19 infection, leukopenia, neutropenia, and mild thrombocytopenia.

## Case presentation

We herein report a rare presentation of TEC in a 2 years old male patient who suffered from acute asthenia and paleness two weeks after Covid19 infection. Patient was admitted to the emergency department of AORN Santobono Pausillipon hospital where a blood count, a Covid-19 PCR test, a biochemical profile, including iron status, were performed. Testing showed Hb level as 4.6 g/dl (with MCV 75 fl. and Hct 12.9%) and therefore, patient underwent transfusion with 150 ml of packed red blood cells group 0- and was successively transferred to our facility. During hospitalization at our center, patient exhibited fair overall clinical conditions, pallor, asthenia, eupnoeic respiration, regular cardiac activity, and non-palpable hypochondriac organs.

**Fig. 1:**
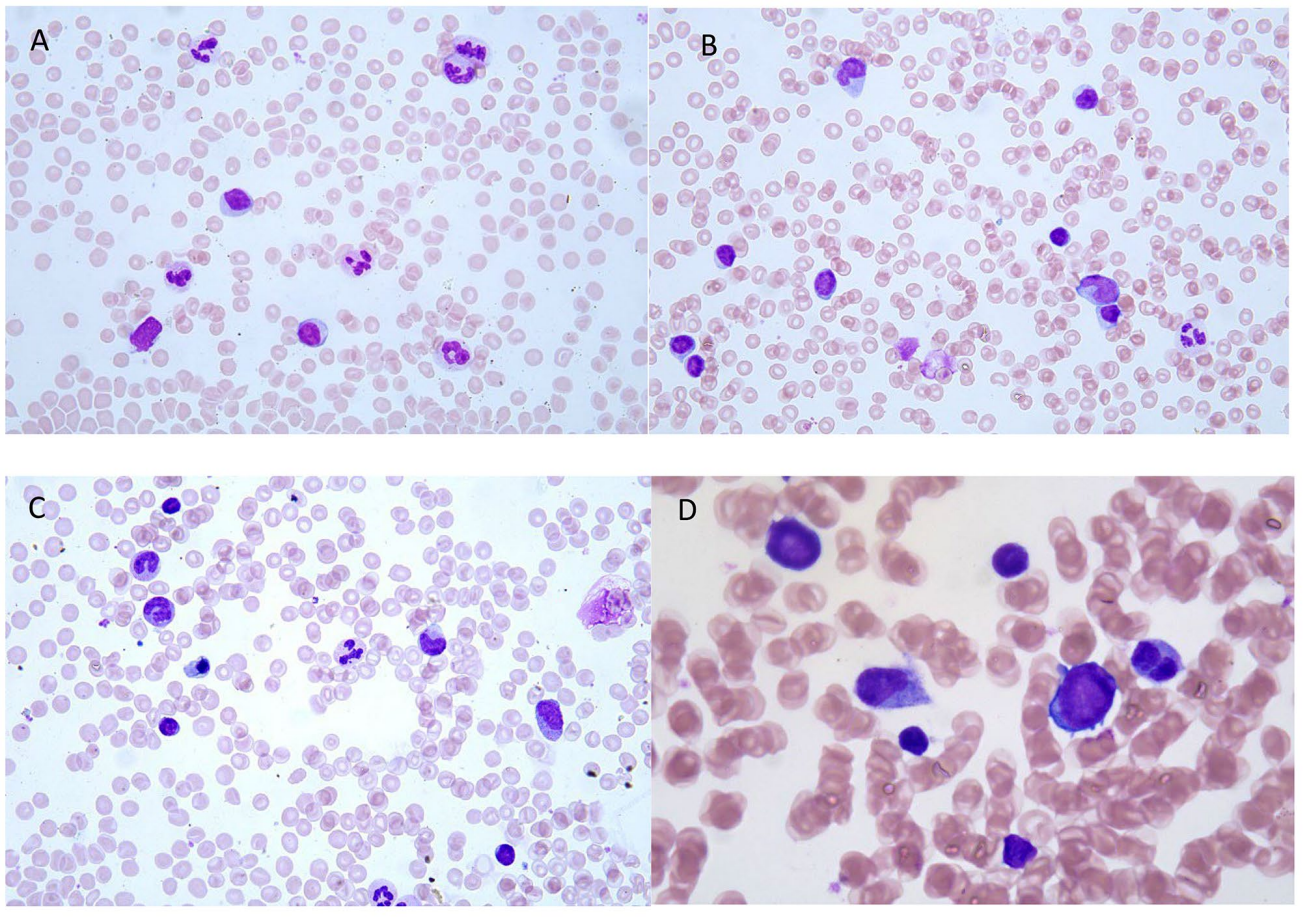
**A.B.C.D**. Bone marrow smear (May Grunwald-Giemsa Staining) shows an erythroid hypoplasia with an almost exclusive detection of late erythroid precursor accounting for 8% of all bone marrow cells. Granuloblastic lineage is normally represented at all stages. Lymphocites are increased. The megakaryocytic series was morphologically normal

**Fig. 2:**
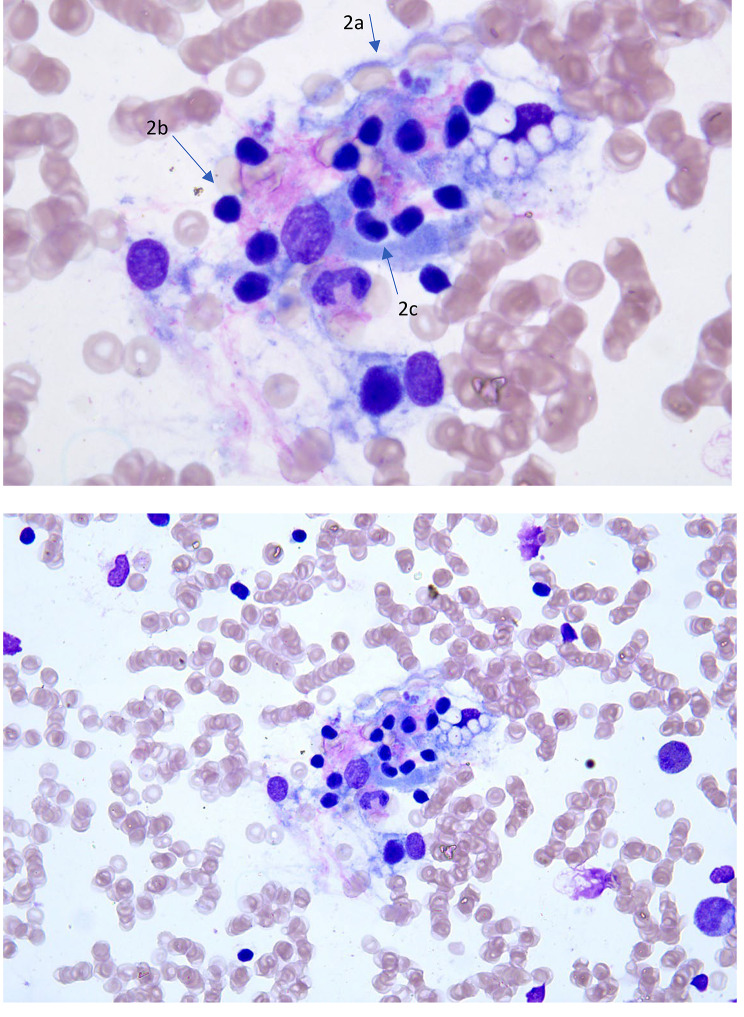
Findings of hemophagocytosis in the same bone marrow smear. The imagine shows phagocytosis of red blood cells (**2a**), lymphocytes (**2b**) and late erythroid precursor (**2c**) (arrow) by four histocytes

Laboratory work-up revealed Hb 7.6 g/dl, low reticulocyte count (10,000/uL), a mild pericardial effusion, negative IgM/IgG viral panel for the main viruses that can be responsible for erythroblastopenia in childhood (such as Parvovirus B19, Epstein-Barr virus, cytomegalovirus, human herpes virus type 6, and echovirus) and complete absence of erythroblasts and signs of erythrophagocytosis at the bone marrow needle aspiration [Figs. 1 and 2], resembling morphological signs such as in hemophagocytic lymphohistiocytosis (HLH).

Given the anamnestic data of Sars-Cov2 infection two weeks before the onset of the clinical manifestations and the absence of clinical signs related to Pediatric HLH [12], a condition already described in adults’ patients affected by COVID19 [13–15], a transient COVID19-driven erythroblastopenia was suspected. Moreover, the Ab anti SarsCov-2 serological tests reported an IgG value of 260 BAU while the hemoglobin electrophoresis showed an HbF value of 1.1% HBF. Furthermore, the erythropoietin value was 55.4 mIU/ml (2.6–18.5 mIU/ml) highlighting a reduced central erythropoiesis. The direct and indirect Coombs tests both yielded negative results. All these findings pointed towards the identification of the Covid 19 infection as a plausible cause of the erythroblastopenia. In line with diagnosis, after 7 days, patient’s laboratory assessments showed a clear improvement of the clinical conditions and the laboratory findings (Hb 12.8 g/dl, 150.000/ul reticulocytes) confirming the transient nature of erythroblastopenia. During additional follow up, anemia resolved, and patient was eventually dismissed from our hematology department.

## Discussion and conclusion

Sars-Cov-2 infection, beyond classic respiratory manifestations, may lead to hematological disorders such as thrombocytopenia [3], lymphopenia [4], neutropenia [5] and disseminated intravascular coagulation [6]. Patient described in our case-report suffered of a severe acute anemia related to a pure red cell aplasia. The main diagnosis suspected before the resumption of the erythropoiesis were TEC and Blackfan Diamond anemia (DBA). The latter, that can be isolated [16] or syndromic [17], was de-prioritized based on the findings of normocytic anemia, age of the patient (2 years-old) and erythropoietin value (55.4 mIU/ml), not as expected in a patient with DBA. Nevertheless, is not so rare that that DBA could outset as a normocytic as well as macrocytic anemia [18]. Moreover, the impossibility to obtain an erythropoietin value prior the hospitalization couldn’t guarantee the real trend of this laboratory finding. TEC, instead, typically occurs due to an immune-mediated mechanism, where the body’s immune system targets and destroys erythroblasts, the precursor cells of red blood cells, leading to a temporary halt in red blood cell production [1]. The specific pathophysiology seems to rely on a massive activation of suppressor T-cells and interferon secretion leading to the production of a suppressive bone marrow microenvironment [19]. While the exact trigger for this immune response is not fully understood, viral infections, as theoretically Covid-19, have been implicated as potential precipitating factors [20].

Current evidence shows that COVID-19 pathophysiology might be mainly driven by a spatiotemporal immune deregulation [21] related to both acute and secondary clinical manifestations.

Current scientific literature shows that anemia may be present heterogeneously in pediatric patients affected by Covid-19 infection, ranging from aplastic [22] to hemolytic [23]. Additionally, in some cases, the infection may exacerbate existing conditions, such as hereditary spherocytosis, wherein the infection may exacerbate hemolysis, thus necessitating blood transfusion [24].

On the same spectrum of immune-related COVID-19 events, multisystem inflammatory syndrome in children (MIS-C), arises because of a dysregulated immune response following Covid-19 infection [25]. The virus indeed can trigger an exaggerated immune response in some individuals, leading to systemic inflammation affecting various organs, including the hematologic system [25]. This inflammatory cascade can disrupt normal hematopoiesis, the process of blood cell formation, leading to anemia among other hematologic abnormalities [26].

In both conditions, the immune response triggered by Covid-19 plays a central role in the development of anemia. However, the specific mechanisms underlying, and the possible inter-links connecting each condition may vary, necessitating tailored diagnostic and therapeutic approaches for optimal management.

In conclusion, a TEC linked to a SARS-CoV‐2 infection has never described in literature, but, according to our findings, should be considered in all the patients with transient erythroblastopenia without congenital red blood cell abnormalities and serology negative for major infections associated with TEC. This condition might be considered in the same spectrum of MIS-C and the inter-links among the two clinical manifestations, as well as a potential interdependence among them, should be considered in the future.

## Data Availability

Data sharing is not applicable to this article as no datasets were generated or analysed during the current study.
